# Pre-colonization with the fungus *Candida glabrata* exacerbates infection by the bacterial pathogen *Clostridioides difficile* in a murine model

**DOI:** 10.1128/msphere.00122-23

**Published:** 2023-06-26

**Authors:** Jesús A. Romo, Makenzie Tomihiro, Carol A. Kumamoto

**Affiliations:** 1 Department of Molecular Biology and Microbiology, Tufts University, Boston, Massachusetts, USA; University of Georgia, Athens, Georgia, USA

**Keywords:** *Candida glabrata*, *Clostridioides difficile*, polymicrobial interactions, biofilms, gastrointestinal infections, colonization

## Abstract

The contributions of commensal fungi to human health and disease are not well understood. *Candida* species such as *C. albicans* and *C. glabrata* are opportunistic pathogenic fungi and common colonizers of the human intestinal tract. They have been shown to affect the host immune system and interact with the gut microbiome and pathogenic microorganisms. Therefore, *Candida* species could be expected to play important ecological roles in the host gastrointestinal tract. Previously, our group demonstrated that pre-colonization of mice with *C. albicans* protected them against lethal *C. difficile* infection (CDI). Here, we show that mice pre-colonized with *C. glabrata* succumbed to CDI more rapidly than mice that were not pre-colonized suggesting an enhancement in *C. difficile* pathogenesis. Further, when *C. difficile* was added to pre-formed *C. glabrata* biofilms, an increase in matrix and overall biomass was observed. These effects were also shown with *C. glabrata* clinical isolates. Interestingly, the presence of *C. difficile* increased *C. glabrata* biofilm susceptibility to caspofungin, indicating potential effects on the fungal cell wall. Defining this intricate and intimate relationship will lead to an understanding of the role of *Candida* species in the context of CDI and novel aspects of *Candida* biology.

**IMPORTANCE:**

Most microbiome studies have only considered the bacterial populations while ignoring other members of the microbiome such as fungi, other eukaryotic microorganisms, and viruses. Therefore, the role of fungi in human health and disease has been significantly understudied compared to their bacterial counterparts. This has generated a significant gap in knowledge that has negatively impacted disease diagnosis, understanding, and the development of therapeutics. With the development of novel technologies, we now have an understanding of mycobiome composition, but we do not understand the roles of fungi in the host. Here, we present findings showing that *Candida glabrata*, an opportunistic pathogenic yeast that colonizes the mammalian gastrointestinal tract, can impact the severity and outcome of a *Clostridioides difficile* infection (CDI) in a murine model. These findings bring attention to fungal colonizers during CDI, a bacterial infection of the gastrointestinal tract.

## INTRODUCTION

The contributions of commensal fungi to human health and disease are not fully understood. *Candida* species such as *C. albicans* are opportunistic pathogenic fungi and common colonizers of the human intestinal tract (1 - 3). They have been shown to affect the host immune system (4 - 8), and interact with the gut microbiome and pathogenic microorganisms (9 - 14). Therefore, *Candida* species could be expected to play important ecological roles in the host gastrointestinal tract. Our group’s demonstration that pre-colonization of mice with *C. albicans* protected them against lethal *Clostridioides difficile* infection (CDI) (15) is consistent with this notion. *C. difficile*, a common intestinal bacterium, causes potentially life-threatening infections by secreting toxins, which leads to intestinal cell damage and cell death (16 - 20). CDI is known to be highly dependent on the state of a host’s microbiome, including the presence or absence of *C. albicans* (21, 22), and therefore broad spectrum antibiotic treatment is a main pre-disposing factor (23). Interestingly, *C. difficile* directly inhibits *C. albicans* filamentation (24), showing that the bacterium can impact fungal biology. Antibiotic treatment has been shown to increase fungal populations, including *C. albicans*, in the gastrointestinal tract of patients and in murine models (21, 22). An expansion of fungi after antibiotic treatment and the pre-disposition to *C. difficile* infection create an environment conducive to fungal-bacterial interactions.

Most of the published microbiome studies on CDI have focused on identifying the relative enrichment of bacterial taxa. A recent study showed strong enrichment of fungi only in patients with CDI, but not in negative control patients with diarrhea (25). However, studies considering fungi in CDI have produced conflicting results with some describing either negative (17, 26, 27) or positive (28, 29) correlations between *C. albicans* colonization and CDI in patients. Additionally, a very small, single institutional, retrospective study, suggested that antifungal use increased the risk of CDI (30). More recently, Cao and co-workers conducted two studies analyzing the human mycobiome in the context of CDI with the goal of developing novel CDI diagnostic tools. They found that patients with CDI exhibit a lower fungal biodiversity and there appears to be a negative correlation between *Candida* and *Saccharomyces* with the *Candida*-to-*Saccharomyces* ration significantly increased in CDI patients (31, 32). The differences in observations and results from these multiple studies could be due to the many differences (e.g., previous medical history) in the patient populations studied. Additionally, *C. albicans* has significant effects on bacterial population diversity after antibiotic treatment in murine models (33), suggesting that it is able to influence microbiome recovery, which could impact CDI. Therefore, fungi are of high importance to CDI, yet highly understudied.

The effects of *C. difficile* are highly dependent on the gastrointestinal microbiota composition of the host (34 - 36). A growing number of studies have investigated the role of the bacterial members before and during infection (37 - 40). These studies have identified bacterial species that can be antagonistic (41, 42) or synergistic (43) to *C. difficile*. Conversely, the role of the fungal components of the microbiota, the mycobiome, is relatively unknown. Previously, our group showed that antibiotic-treated mice pre-colonized with the opportunistic pathogenic fungus *C. albicans* were protected from a fatal *C. difficile* infection (15). This protection was conferred, at least in part, by enhanced IL-17 production stimulated by *C. albicans*.

Here, we focused on the non-filamentous yeast, *C. glabrata* (recently re-classified as *Nakaseomyces glabrata*). *C. glabrata* is an opportunistic pathogenic yeast whose role in the host has been significantly less studied (44 - 46) and is the second most commonly isolated *Candida* from patients (47 - 50). Importantly, *C. glabrata* was recently named as a fungal pathogen of high priority by the World Health Organization (WHO) (51), highlighting its importance as a pathogen. The contributions or impacts of *C. glabrata* as a member of the gut microbiome are not well understood. Here, we show that pre-colonization of antibiotic treated mice with *C. glabrata* exacerbates CDI, suggesting that *C. glabrata* occupies a distinct ecological niche from *C. albicans*.

## RESULTS

### *C. glabrata* pre-colonization exacerbates *C. difficile* infection in a murine model

To further investigate the role of fungal colonizers during *C. difficile* infection, we asked whether *C. glabrata* plays a distinct role in the gastrointestinal tract. A murine model of CDI was coupled to a murine model of fungal gastrointestinal colonization (Fig. 1A). This methodology has been recently described in detail (52). Briefly, 5-week-old female C57BL/6 mice were co-housed and provided cefoperazone in their drinking water. At the end of 10 days, mice were switched to water without cefoperazone and the day after this transfer, given PBS or *C. glabrata* BG2 via oral inoculation. Mice were administered clindamycin intraperitoneally and fed *C. difficile* UK1 spores (3, 5 × 10^5^ spores per mouse) on the next day. The presence of *C. glabrata* exacerbated CDI leading to earlier disease onset, increased severity, and mortality (Fig. 1B). Mice pre-colonized with *C. glabrata* and challenged with *C. difficile* began succumbing 2 days earlier compared to the *C. difficile* only group (*P* = 0.0018; log rank test) (Fig. 1B). Importantly, mice pre-colonized only with *C. glabrata* did not exhibit any visible disease.

**Figure:**
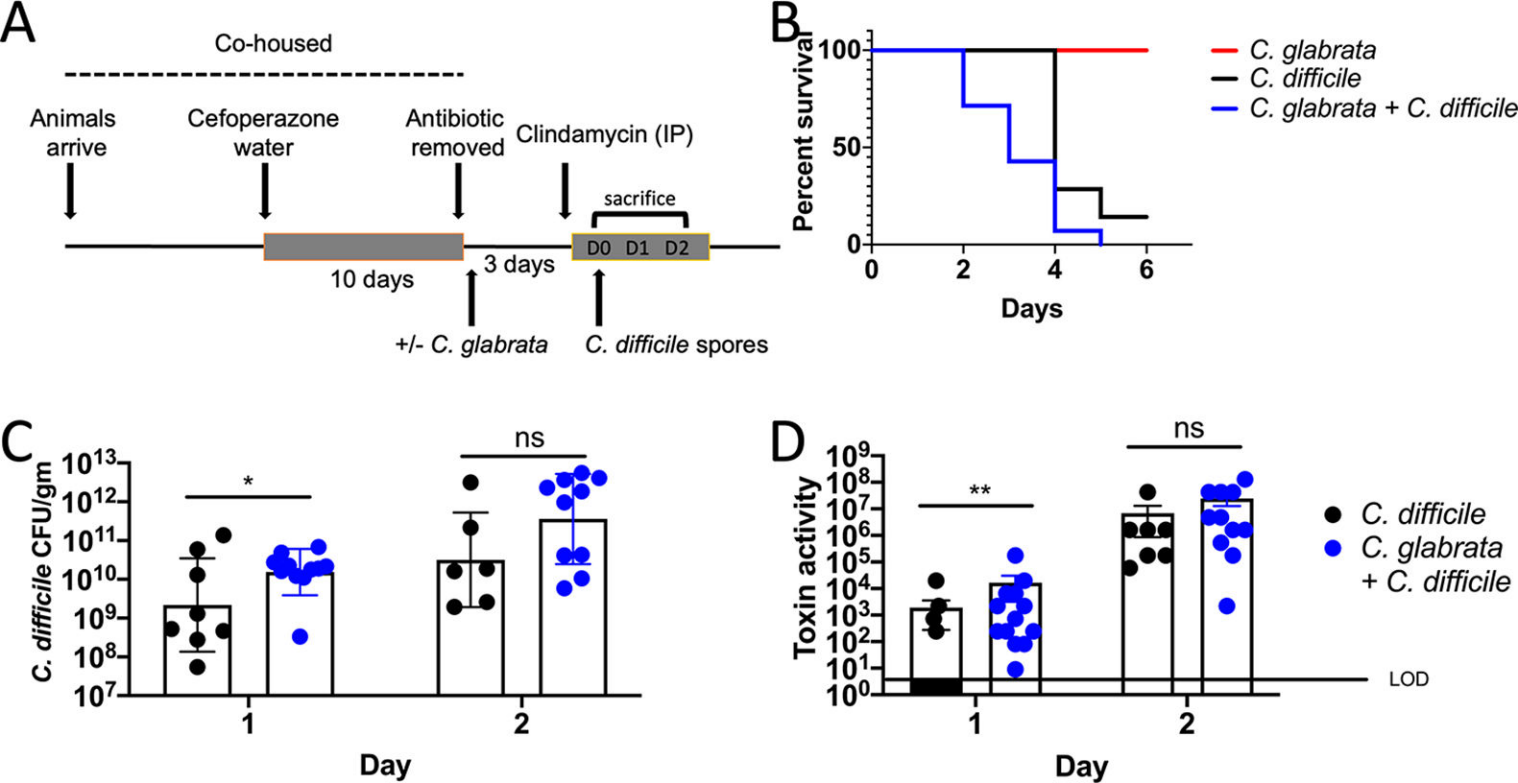
**FIG 1** *C*. *glabrata* pre-colonization exacerbates CDI in a murine model. Antibiotic treatment and infection schema is shown in (**A**). The C57BL/6 mice were co-housed in a large cage and received antibiotics as described in Materials and Methods (cefoperazone 10 days). Mice were then transferred to standard size cages, some mice were fed *C. glabrata*, and all mice were switched to water without antibiotics for 2 days. Mice received clindamycin intraperitoneally followed by *C. difficile* spores (3, 5 × 10^5^ spores of strain UK1, a NAP1/027/BI human epidemic strain) by oral inoculation on the next day. (**B**) Survival of the mice was monitored for 6 days post-inoculation with spores (*C. glabrata*, *n* = 12; *C. difficile*, *n* = 12; *C. glabrata* and *C. difficile*, *n* = 12 log rank test *P* = 0.0018). (**C**) *C. difficile* bacteria were enumerated by plating the homogenized cecum contents collected from mice sacrificed on day 1 or 2 post-inoculation. Homogenates were plated on a TCCFA medium. Bar indicates the geometric mean. Mann-Whitney *P*-values are displayed above each set. *, *P* = 0.0112. (**D**) *C. difficile* toxin activity in the cecum contents from the mice sacrificed on day 1 or 2 post-inoculation was measured using a cell-rounding assay. The inverse of the greatest dilution that yielded 100% cell rounding is plotted. Mann–Whitney *P*-values are displayed above each set. **, *P* = 0.007 combined results of three experimental trials.

A separate group of *C. glabrata* only mice were followed for 28 days. These mice continued to be colonized with *C. glabrata* (10^7^ cfu/gm in fecal pellets) and gained weight normally (Fig. S1). These findings show that *C. glabrata* can colonize mice for extended periods of time without apparent detrimental effects to the host.

Mice pre-colonized with *C. glabrata* contained higher cecal *C. difficile* CFU 1 day after *C. difficile* inoculation compared to *C. difficile* only mice (Fig. 1C). Moreover, all mice (*n* = 12) pre-colonized with *C. glabrata* contained detectable toxin in their ceca 1 day after *C. difficile* infection compared to only four mice (*n*=12) with detectable toxin from the *C. difficile* only group (Fig. 1D). These findings show that *C. glabrata* colonization leads to CDI exacerbation, which could be due in part to earlier toxin production. Importantly, no correlation was observed between CFU, and amount of toxin detected at day 1 or 2 after infection, similar to previous reports (17, 53). Further, no significant differences in cecum tissue histology were detected between the groups at either of the two timepoints (Fig. S2). Thus, in contrast to *C. albicans* (15), *C. glabrata* pre-colonization produces a distinct effect, negatively impacting CDI outcome. The exact mechanisms of this exacerbation are most likely multifactorial.

**Figure:**
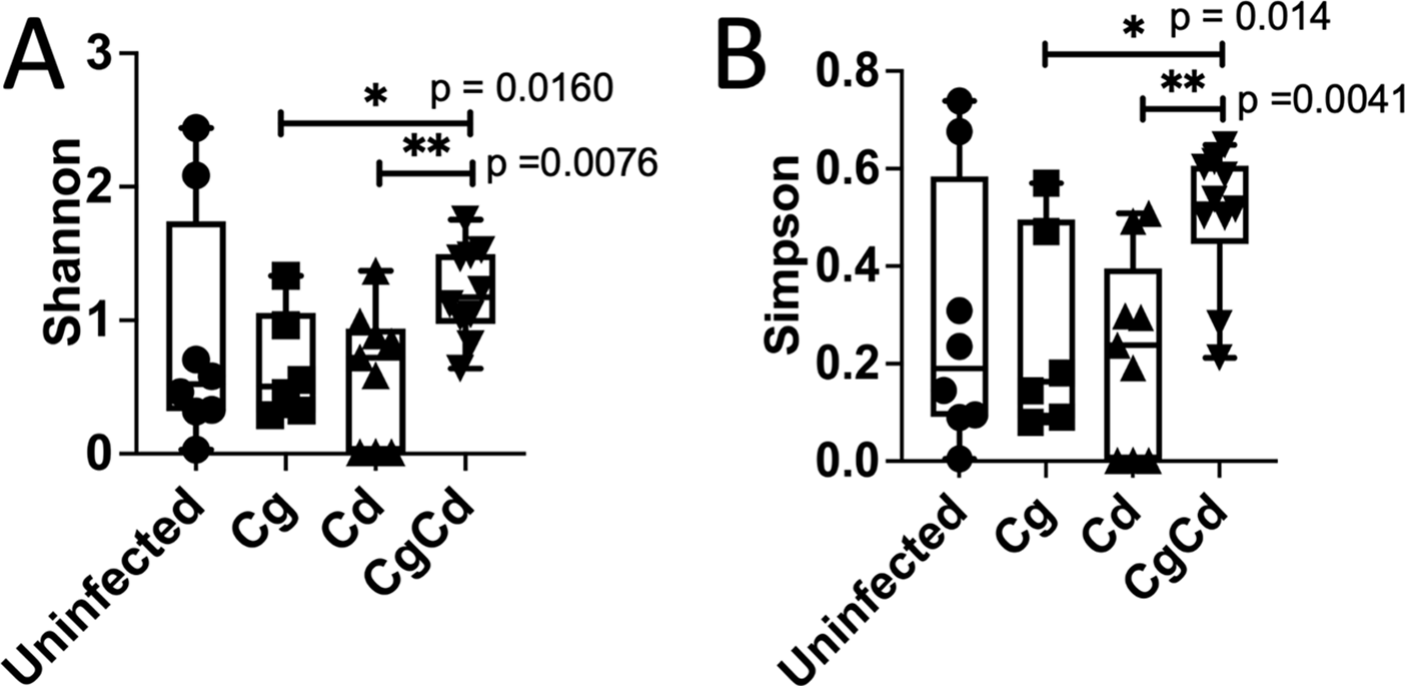
**FIG 2** Cecal microbiota community diversity scores. Ceca were collected from mice after sacrifice 2 days post-*C. difficile* inoculation. Bacterial composition was analyzed by 16S rDNA sequencing followed by QIIME2 analysis. Diversity scores were calculated. Bar represents the mean diversity score for all mice within a group. (**A**) Shannon and (**B**) Simpson. Combined results of three experimental trials. Groups were compared using a Kruskal–Wallis test.

Microbiota composition of the host gastrointestinal tract plays a significant role in preventing CDI by a variety of mechanisms (e.g.*,* limiting access to resources and secretion of inhibitory metabolic products). Indeed, CDI is highly dependent on the host microbiota composition and several bacterial genera have been implicated in antagonistic roles toward *C. difficile* (41, 42). *Candida* species have been shown to influence microbiota reassembly and composition after antibiotic treatment and immunosuppression (22, 54 - 57). Therefore, we characterized the cecal microbiota composition in our murine model to understand its role in disease exacerbation. Mice were divided into uninfected, *C. glabrata* only, *C. difficile* only, and *C. glabrata + C. difficile* cohorts as described above (Fig. 1A). Two days post-inoculation with *C. difficile* spores, mice were sacrificed, and the distal cecum tip was harvested, placed in a collection tube and frozen immediately. Samples were processed as described in Materials and Methods. The composition of the cecal bacterial community was characterized by sequencing the V4 region of the 16S rRNA gene and analyzed using QIIME 2 (2018.8) (58) (Fig. 2 and 3).

**Figure:**
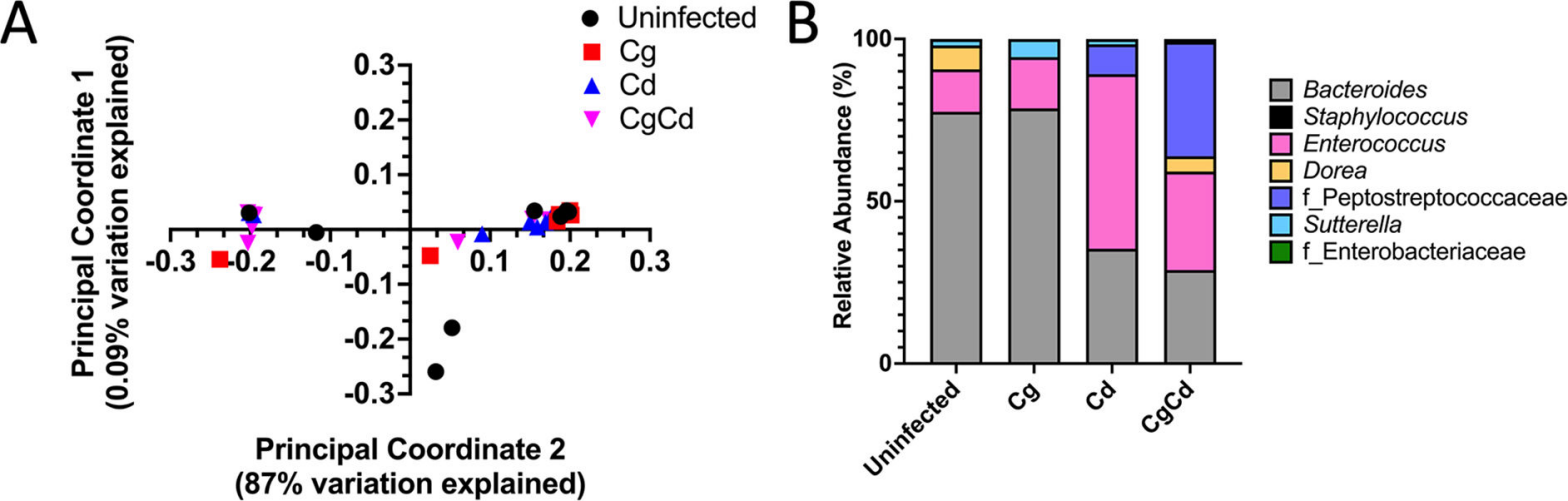
**FIG 3** Analyses of cecal bacterial communities. On day 2 post-inoculation with *C. difficile* spores, mice were sacrificed, and their ceca were removed. Bacterial composition was analyzed by 16S rDNA sequencing followed by QIIME 2. (**A**) Weighted UniFrac distances were used to construct a Principal coordinate analysis. Black circles indicate uninfected, red squares indicate *C. glabrata* only, blue triangles indicate *C. difficile* only, and pink upside-down triangle indicates *C. glabrata* and *C. difficile*. (**B**) Relative abundance of bacterial genera in the cecal microbiota (*n* = 12 mice per group). All genera with median > 0 in at least one group are shown.

Alpha diversity analyses highlighted a significant increase in diversity of the population between the *C. difficile* only and *C. glabrata + C. difficile* groups (Fig. 2A and B), although no significant differences were observed using the Chao1 and Faith’s alpha diversity analyses (Fig. S3). The weighted UniFrac distance between bacterial communities is represented in a principal coordinate analysis (PCoA) (Fig. 3A). The PERMANOVA analysis of these results identified significant differences between *C. difficile* and *C. glabrata + C. difficile* (*P* = 0.016).

To identify possible differences in genera between the groups, the relative abundances of bacterial genera were compared (Fig. 3B). All bacterial genera with a median fraction greater than zero in at least one of the two groups were included in this analysis (Fig. 3B). Due to antibiotic treatment in preparation for the *C. difficile* infection, mice were colonized with relatively few genera. We observed differences in the relative abundance of certain groups. An increase in relative abundance of members of the *Peptostreptococcaceae* family (the family that includes *C. difficile*) and a decrease in the genus *Enterococcus* (Fig. 3B) were observed. Importantly, these results showed comparable trends to findings previously reported in patients (59). Lastly, similar changes were observed in phyla (Fig. S4).

Total levels of bacteria were measured using qPCR and universal 16S rRNA primers as previously described (60). Relative abundance was multiplied by the total level of bacteria per milligram of cecum sample (Fig. S5) to determine absolute abundance in arbitrary units per mg of cecum contents. Absolute abundances of genera in mice with *C. difficile* and either with or without *C. glabrata* were very similar, but a statistically significant increase in the *Peptostreptococcaceae* family (*P* = 0.0057) was observed in mice with *C. difficile* and *C. glabrata* (Fig. S5). These findings suggest that bacterial microbiota composition is not the sole factor impacting disease progression and outcome in this model.

### *C. glabrata* and *C. difficile* form robust polymicrobial biofilms

Both *Candida* species and *C. difficile* have been shown to interact with members of the microbiome (9, 41, 61, 62). Therefore, an additional factor impacting disease exacerbation could be polymicrobial interactions between *C. difficile* and *C. glabrata*. We detected an interaction between *C. difficile* and *C. glabrata* using a co-culturing system focused on biofilms.

A highly robust and replicable fungal biofilm model was adapted to study the effects of *C. difficile* on pre-formed fungal biofilms (Fig. 4). Pre-formed fungal biofilms were used because presumably, *C. glabrata* would be present and established in the human gastrointestinal tract before *C. difficile* was present. *C. glabrata* biofilms were grown in tissue culture media (DMEM, 10% FBS, and 1% NEAA) at 37°C under anaerobic conditions for 24 h before the addition of *C. difficile* as described in Materials and Methods. After the addition of *C. difficile*, biofilms were further incubated for 24 h. The addition of *C. difficile* to a *C. glabrata* biofilm led to a significant enhancement in overall biofilm biomass and matrix compared to either *C. difficile* or *C. glabrata* mono-microbial biofilms (Fig. 4A and B). Importantly, biomass from the co-culture was greater than the biomass predicted due to an additive effect. This observation is significant as both organisms formed weak biofilms independently under the same conditions at 24 h (63). Furthermore, no significant change in the metabolic activity of *C. glabrata* was observed when *C. difficile* was present (Fig. 4C). Metabolic activity was measured using the XTT assay as previously described (64, 65). To distinguish between the metabolic activity of both organisms, plates were taken out of the anaerobic chamber and washed multiple times with aerobic PBS to reduce *C. difficile* viability. *C. difficile* mono-microbial biofilms treated the same way did not reduce XTT (not shown). Additionally, plating of *C. glabrata* cells from the biofilms on YPD showed that there was no significant difference in CFU between *C. glabrata* only and *C. glabrata* with *C. difficile* (Fig. 4D; Fig. S6). These results suggest that the presence of *C. difficile* leads to an enhancement in biofilm formation independent of *C. glabrata* cell number. Moreover, these results show that interaction with *C. difficile* was not cytotoxic to *C. glabrata*. Similar experiments were conducted to assess *C. difficile* viability except BHIC-TA was used as the plating medium and the plates were incubated anaerobically. There were no significant differences in CFU from *C. difficile* only biofilms or polymicrobial biofilms and as with *C. glabrata*, the enhancement in biofilm biomass was independent of *C. difficile* CFU (Fig. 4E). A similar phenotype of biofilm enhancement was observed when introducing *C. difficile* to biofilms of two distinct oral clinical isolates of *C. glabrata* (Fig. S7) (66). Lastly, *C. difficile* toxin production was assessed in mono or polymicrobial biofilms (Fig. S8). Toxin titers were similar between biofilms, and thus, *in vitro*, the presence of *C. glabrata* did not enhance toxin production. These findings show that these two organisms form a highly robust polymicrobial biofilm *in vitro* without impacting each other’s viability.

**Figure:**
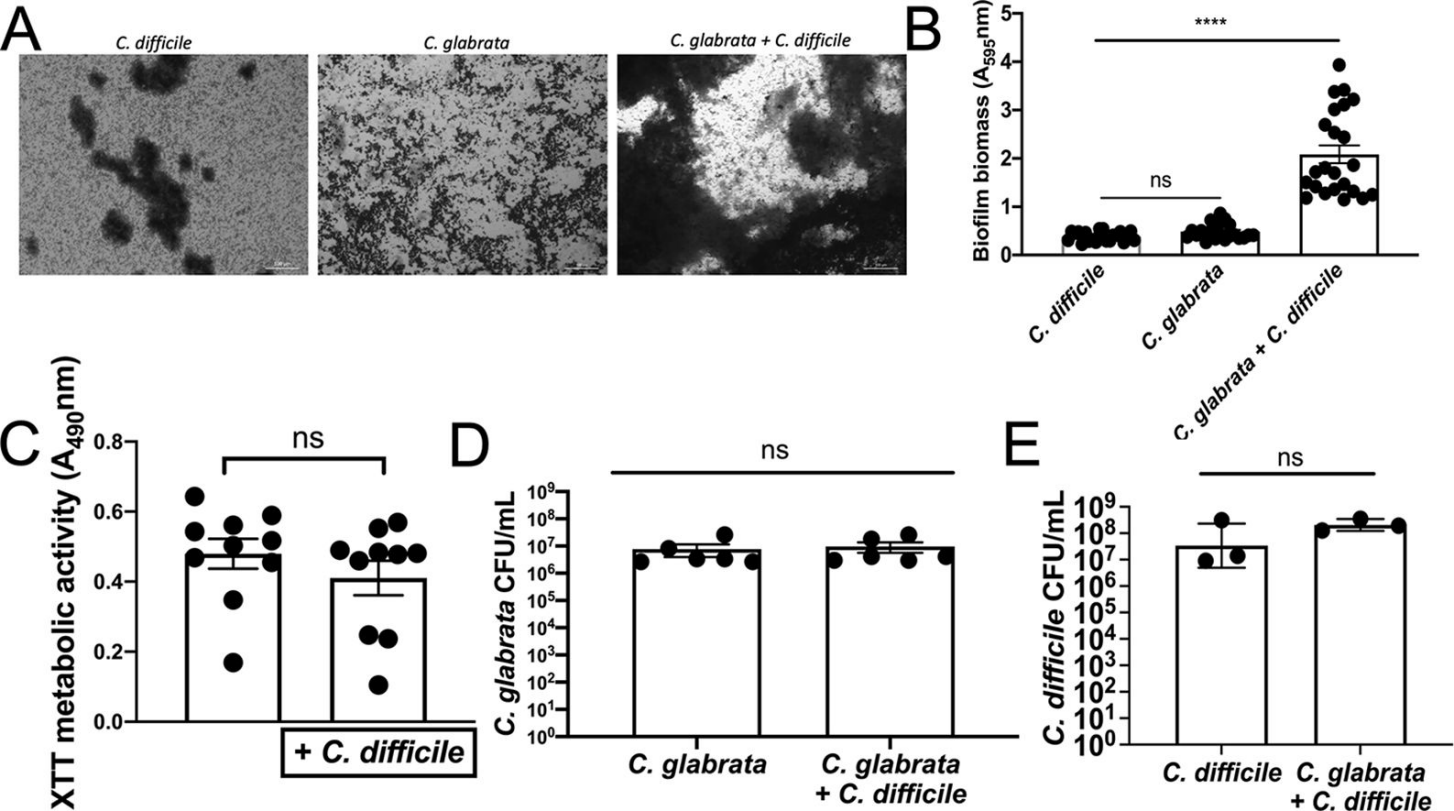
**FIG 4** *C. glabrata* and *C. difficile* form a robust polymicrobial biofilm. *C. glabrata* biofilms were formed anaerobically for 24 h followed by the addition of *C. difficile* and further 24 h incubation. (**A**) Crystal violet staining of a 24 h *C*. *difficile* biofilm (left), 24 h *C*. *glabrata* biofilm (center) or polymicrobial biofilm (right). (**B**) Biofilm biomass quantification. (**C**) Quantification of metabolic activity by XTT assay. (**D**) *C. glabrata* biofilm CFU in the presence and absence of *C. difficile*. (**E**) *C. difficile* biofilm CFU in the presence and absence of *C. glabrata*. Mann–Whitney *P*-values are displayed above each set. ****, *P* < 0.0001. Representative results of four experimental trials with > 20 technical replicates.

### *C. difficile* sensitizes *C. glabrata* to the cell wall targeting agent caspofungin and impacts cell wall related processes

Polymicrobial biofilms are highly resistant to antimicrobial treatments (67). We sought to characterize the antifungal sensitivity profile of *C. glabrata* in polymicrobial biofilms with *C. difficile* using the model described above. The XTT assay was used as a readout for *C. glabrata* metabolic activity as described in Materials and Methods. The presence of *C. difficile* significantly sensitized *C. glabrata* to the cell wall targeting antifungal caspofungin at most concentrations tested (Fig. 5A). This increase in susceptibility was not observed with other antifungals tested (Fig. S9) suggesting that *C. difficile* impacts *C. glabrata* cell wall biology.

**Figure:**
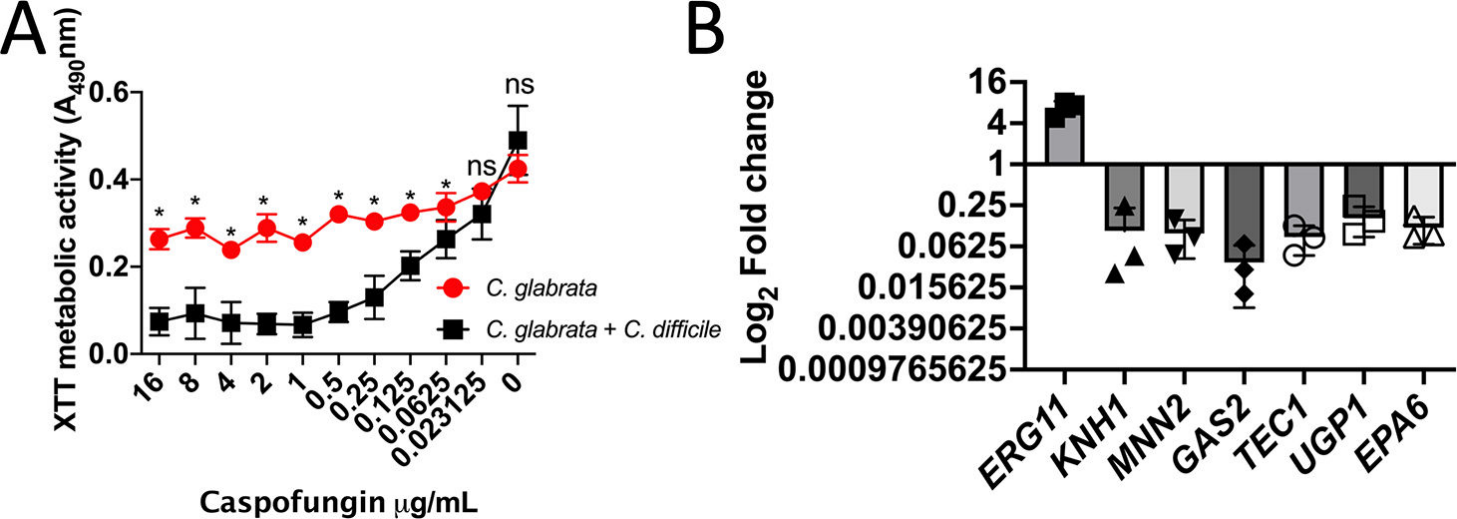
**FIG 5** *C. difficile* impacts the *C. glabrata* cell wall. Polymicrobial biofilms of *C. glabrata* and *C. difficile* were formed anaerobically. (**A**) Biofilms were grown anaerobically and exposed to the antifungal caspofungin anaerobically in a dose response assay and the impact on the biofilm was quantified using the metabolic XTT assay. (**B**) RNA was isolated from the polymicrobial biofilms and expression of *C. glabrata* genes in the presence of *C. difficile* relative to *C. glabrata* alone was measured using qRT PCR (3 experimental trials).

Additionally, a dose response assay was conducted to measure susceptibility to the antibiotic vancomycin. The PrestoBlue reagent was utilized to distinguish *C. difficile* metabolic activity from that of *C. glabrata*. The presence of *C. glabrata* increased *C. difficile* metabolic activity (no vancomycin, *P* = 0.0004) suggesting an enhancement in biofilm formation (Fig. S10). Moreover, the presence of *C. glabrata* appeared to decrease *C. difficile*’s susceptibility to vancomycin (Fig. S10).

To better understand the impact of *C. difficile* on *C. glabrata* cell wall biology, transcription of genes whose products are involved in a variety of cell wall and biofilm related processes was measured (Fig. 5B). We focused on *ERG11*, which encodes Lanosterol 14-alpha-demethylase, a key enzyme in ergosterol biosynthesis (68), *KNH1* encoding a product important in β1,6-glucan synthesis (69), *MNN2* encoding an α-1,2-mannosyltransferase important for the glycosylation of cell wall components (70), and *GAS2* encoding a glycosylphosphatidylinositol (GPI)-anchored cell surface protein that has been shown to be a regulator of β1,3-gulcan and important in drug treated biofilms of *C. glabrata* (71 - 73). Further, *TEC1* encodes a product important for biofilm formation and adaptation to low pH stress in *C. glabrata* (74) and *UGP1* encodes a Putative UDP-glucose pyrophosphorylase involved in the β1,6-gulcan biosynthetic process (75, 76). We also measured the expression of *EPA6*, a gene whose product is one of the key adhesins in *C. glabrata* and is important for biofilm formation (77 - 79). While *ERG11* expression was upregulated (8-fold), the rest of these cell wall and biofilm related genes were significantly downregulated in the presence of *C. difficile*. These findings support the model that *C. difficile* impacts fungal cell wall biology. These interactions could have significant implications for CDI progression in the gastrointestinal tract.

## DISCUSSION

While the contributions of commensal fungi during bacterial infections are not well understood, fungi have been implicated in a variety of polymicrobial interactions that could significantly impact host health (9, 55, 80 - 83) including those involving CDI (15, 24, 25, 28, 29, 60, 84 - 86). More recently, Santus and co-workers showed that *Salmonella enterica* can utilize commensal and dietary fungal siderophores which promote *Salmonella* colonization (87). This study highlights the complexity of *trans* kingdom polymicrobial interactions and underscores the critical need to characterize the role of the mycobiome in host health and disease.

Here, we describe the use of a murine model of CDI coupled with a fungal colonization model to characterize the impact of the opportunistic pathogenic fungus, *C. glabrata* during CDI. We show that pre-colonization of mice with *C. glabrata* exacerbates CDI leading to more rapid mortality. Moreover, the co-colonized mice harbored higher levels of *C. difficile* (detected by plating for CFU and sequencing) and more detectable toxin compared to mice that only carried *C. difficile* at an early time point. These two factors probably contribute to disease exacerbation, but exacerbation is likely multifactorial as toxin production does not correlate with disease outcome in CDI (17, 53). Moreover, in our model, CFU did not correlate with toxin production at either day 1 or 2 (not shown), further highlighting the complexity of the system. Previously, Jawhara and co-workers utilized a dextran sulfate sodium (DSS)-induced colitis murine model to characterize the contribution of *C. glabrata* cell wall components during gastrointestinal colonization (88). In this study, *C. glabrata* was able to colonize the murine gastrointestinal tract without causing visible disease, which was also seen in our studies. When DSS was fed to mice in their drinking water, 25% of the *C. glabrata* colonized animals died, suggesting that *C. glabrata* can exploit a colitis environment. This study attributed disease to enhanced colonization and the ability of *C. glabrata* to cause damage in colitis tissues and did not assess dissemination of *C. glabrata* to other tissues. Toxins produced by *C. difficile* induce colitis in the gastrointestinal tract (89 - 94); therefore, it is possible that *C. glabrata* exploits this environment during CDI. While we did not observe an increase in inflammation in the *C. glabrata + C. difficile* compared to the *C. difficile* only group (Fig. 3), β-glucans and chitin in the fungal cell wall have been shown to have anti-inflammatory effects (54, 95) potentially obscuring any additional inflammatory effects in the *C. glabrata + C. difficile* groups.

*C. difficile* infection is highly dependent on the status of the host gastrointestinal microbiome. While we did not identify large changes in overall bacterial microbiota composition in co-colonized mice compared to the other groups, we did identify changes in relative abundance of some taxa including a significantly higher relative abundance of the *Peptostreptococcaceae* family. Moreover, the trends observed in our murine models were similar to those reported in patients previously (59). Interestingly, in the co-colonized mice, we identified a lower relative abundance of the genus *Enterococcus*, a group of organisms that is often enriched in *C. difficile* infections (37, 96). Importantly, Enteroccoci have been shown to be antagonistic to *C. difficile* growth (97), or in more recent studies, enhance *C. difficile* virulence via a nutrient restriction and cross-feeding mechanism (98). The presence of *C. glabrata* may enhance *C. difficile* fitness, allowing it to outcompete other members of the microbiome. The findings from our murine studies suggest that the presence of *C. glabrata* fuels CDI by enhancing early *C. difficile* growth and toxin production.

Utilizing an *in vitro* co-culturing system, we further show that *C. difficile* and *C. glabrata* form robust polymicrobial biofilms compared to their monomicrobial counterparts. Interestingly, while they do not affect each other’s viability, the presence of *C. difficile* impacts the expression of *C. glabrata* genes whose products are involved in cell wall biogenesis and maintenance and sensitizes *C. glabrata* to caspofungin, a cell wall targeting antifungal. These findings suggest that *C. difficile* can directly or indirectly impact fungal cell wall biology. We propose that these findings translate to the gastrointestinal tract via three potential mechanisms. First, *Bacteriodetes* have been shown to utilize yeast mannans, a mechanism that fuels growth in the gastrointestinal tract (99). *C. difficile* could be utilizing a similar strategy. Second, *Candida* species colonizing the gastrointestinal tract mask glucans present in their cell walls to evade immune detection (4, 100 - 102). It is possible that *C. difficile* leads to dysregulation of the fungal cell wall modifying the glucan masking mechanism and eliciting an immune response from the host. These effects may lead to enhanced mortality in our model. Third, *C. difficile* has been shown to form biofilms with other members of the microbiome, which could enhance its survival and proliferation in the host gastrointestinal tract (98, 103). Polymicrobial biofilms with members of the mycobiome such as *C. glabrata* could play a significant role in CDI.

Currently, knowledge of mucosal biofilms in the gastrointestinal tract is limited, but reports suggest that they contribute to colonization resistance, community stability and resilience, food digestion, and drug chemical modifications (104). The role of biofilms in CDI is currently not known. *C. difficile* biofilms were first reported clogging a biliary stent (105) and have begun to receive interest in recent years (103, 106 - 116). Importantly, biofilms have been suggested to serve as reservoirs that could play a role in recurrence of CDI (115), but this has yet to be demonstrated.

It is currently unknown whether this ability to form a polymicrobial biofilm plays a significant role in the mammalian host. Interestingly, biofilms formed by members of the host microbiome, including the fungus *C. parapsilosis*, have recently been shown to harbor *C. difficile* in a gut chemostat model (103). This ability to form polymicrobial biofilms with members of the host microbiome, including fungi, could serve to harbor *C. difficile* and serve as a reservoir for recurrent infections.

Our *in vitro* model used biofilms to study interactions between *C. difficile* and *C. glabrata* and yielded highly interesting findings suggesting a mechanism involving the fungal cell wall. Interestingly, previous studies have reported an increase in virulence of cells involved in a biofilm (117). While fungi and bacteria in the gut may not form true biofilms, it is possible that the cells are in close proximity to each other, potentially allowing them to interact in ways that resemble the interactions detected in biofilms. These possibilities will require further investigation in the future.

Utilizing the murine model presented here will allow the characterization of the role of individual members of the mycobiome and will lead to the development of novel therapeutic interventions for CDI.

## MATERIALS AND METHODS

### Strains and growth conditions

*C. glabrata* strain BG2 (118, 119), a clinical isolate from a vaginitis case that did not respond to fluconazole, was used for all studies (except for Fig. S7). Clinical isolates AE2 and D1 were kindly provided by Dr. Célia F. Rodrigues and described previously by her group (66). Cells were grown at 37◦C in YPD broth [1% yeast extract (BD, Sparks, Maryland, USA cat. 212750), 2% peptone (Difco, Detroit, Michigan, USA cat. 0118-17-0), and 2% glucose (Sigma-Aldrich, St. Louis, Missouri, USA cat. G8270)]for 21–24 h in preparation for mouse inoculation. *C. glabrata* cells were grown at 30°C in YPD broth for 24 h before co-culturing experiments. Enumeration of *C. glabrata* cells was performed by plating diluted samples (from mouse cecum contents or *in vitro* biofilms) on YPD-SA agar medium [YPD agar plus 100 µg/mL streptomycin (Sigma-Aldrich, St. Louis, Missouri, USA cat. S6501) and 50 µg/mL ampicillin (Sigma-Aldrich, St. Louis, Missouri, USA cat. A9518)] and incubated for 2 days at 37°C. The *C. difficile* NAP1/027/BI human epidemic strain, UK1 (38), was used for all studies. Cultures were grown in pre-reduced TY broth (3% tryptose, 2% yeast extract, 0.1% thioglycolate, pH 7.4) (60). Spores were isolated as previously described (120, 121)without gradient purification. *C. difficile* vegetative cells and spores in the extracts from mice were enumerated by plating the samples on pre-reduced TCCFA plates [Taurocholate (Calbiochem, San Diego, California, USA cat. 580217), cycloserine (Sigma-Aldrich, St. Louis, Missouri, USA cat. C6880), cefoxitin (Sigma-Aldrich, St. Louis, Missouri, USA cat. C4786), fructose (Macron Fine Chemicals, Center Valley, Pennsylvania, USA cat. 7756-12)] (52) and incubated at 37°C for 2 days in an anaerobic chamber. Samples were heated at 60°C for 10 min, followed by plating on pre-reduced TCCFA plates for spore enumeration.

### Murine model of gastrointestinal colonization and CDI

All the experiments using animals were done in compliance with the NIH Guide for the Care and Use of Laboratory Animals and Tufts University IACUC guidelines. Animal experimentation was approved by the Tufts Institutional Animal Care & Use Committee, 10/08/2018, protocol number B2018-84. The murine model was recently described in detail (52). Briefly, 5-week-old female C57BL/6 mice (Jackson Laboratory, Bar Harbor, Maine, USA) were co-housed in a large cage (24″ × 17″) for 20 days total. After 10 days, mice were treated with the antibiotic cefoperazone (Sigma-Aldrich, St. Louis, Missouri, USA cat. C4292, 0.5 gm/L) in drinking water for the remaining 10 days of co-housing. At the end of the cefoperazone treatment, all mice were tested and shown to be negative for cultivable fungi on YPD-SA agar medium [YPD agar plus 100 µg/mL streptomycin (Sigma-Aldrich, St. Louis, Missouri, USA cat. S6501) and 50 µg/mL ampicillin (Sigma-Aldrich, St. Louis, Missouri, USA cat. A9518)] incubated for 2 days at 37°C. After 20 days of co-housing, all mice were transferred from the large cage to standard sized cages, housing four mice per cage and switched to water without cefoperazone. Mice were then given *C. glabrata* orally (5 × 10^8^ cells in 25 µL) or PBS. For *C. glabrata* colonization experiments (*n* = 8), mice were allowed to rest for 28 days. *C. glabrata* colonization was measured as described previously (52). Briefly, 3–4 fecal pellets were collected from each animal into a pre-weighed tube containing PBS. The tube was weighed after collection. Pellets were then homogenized using a microtube vortex, serially diluted, and plated for CFU on YPD-SA. For the *C. difficile* infection experiments (*n* = 12), mice were treated as above with cefoperazone and orally inoculated with *C. glabrata* or PBS, then allowed to rest for 2 days. All the mice used in these experiments were shown to be negative for *C. difficile* colonization prior to inoculation with spores by collecting fecal pellets and plating on pre-reduced TCCFA. After the 2 days of rest, they were administered clindamycin intraperitoneally (Sigma-Aldrich, St. Louis, Missouri, USA cat. C5269) (10 mg/kg). The following day, mice were orally inoculated with *C. difficile* spores (3, 5 × 10^5^ spores per mouse). Mice were then monitored for survival or sacrificed at 1 or 2 days after *C. difficile* inoculation to assess disease progression. The mice were weighed daily at the same time of day and sacrificed when moribund. Mice were considered moribund if they exhibited severe signs of illness (extreme inactivity, hunched posture, ruffled fur). If their weight loss exceeded 20%, they were also sacrificed. To ascertain if *C. glabrata* was able to disseminate during *C. difficile* infection, murine liver, kidneys, and tongues were collected. The tissues were macerated, diluted, and plated on YPD-SA for *C. glabrata* and BHI for aerobic bacteria. The survival data show the combined results of the mice from multiple experimental trials. The relative weights were compared using the *t* test. Survival was compared using the log rank test.

### *C. difficile* toxin titer assay

African green monkey kidney epithelial cells (Vero cells) (ATCC CCL-81) were seeded in 96-well microtiter plates at a concentration of 4.3 × 10^3^ cells per well in DMEM (Corning Cell Gro, Corning, New York, USA cat. MT10-013CV) with 10% heat-inactivated fetal bovine serum (Atlanta Biologicals S11150) and 1% MEM non-essential amino acids (ThermoFisher Scientific, Waltham, MA, USA cat. 11140076) and allowed to adhere for 24 h at 37°C in 5% CO_2_ (52, 60). Cecum contents from mice were weighed and diluted 1:10 with DMEM with 10% FBS and 1% MEM non-essential amino acids. Serial threefold dilutions in DMEM with 10% FBS and 1% MEM non-essential amino acids, were added to adhered Vero cells and incubated for 24 h at 37°C in 5% CO_2_. Cells were scored visually at 10× magnification for cell rounding at 24 h. The toxin titer is defined as the inverse of the greatest dilution that resulted in 100% cell rounding.

### Histology

One or 2 days post-inoculation with *C. difficile*, cecum tissue was collected from mice and fixed in buffered formalin and processed for staining with Hematoxylin and Eosin (H&E) by the Tufts Comparative Pathology Core facility. Scoring was conducted at 10× magnification on 30–40 fields for each tissue of view by a blinded investigator. The scores were calculated by dividing the number of fields showing infiltration/inflammation by the total number of fields examined for that tissue. Each point on the graph represents an individual mouse. A non-parametric *t*-test was used to compare the groups.

### Microbiota analyses

Two days post *C. difficile* inoculation, mice were sacrificed, and the distal tip of the cecum was immediately frozen on dry ice. Microbial DNA was extracted using the QIAaMP PowerFecalPro DNA Kit (Qiagen, Hilden, Germany cat. 12830-50) according to the manufacturer’s protocols. The recommended additional steps to ensure optimal yield of DNA from Gram-positive bacterial was also followed. Libraries were prepared and sequenced as previously described (122). Briefly, the V4 region of the 16S rRNA was amplified via PCR with 515F and 806R primers including adapters for Illumina sequencing and 12-mer Golay barcodes for multiplexing. Two hundred fifty base pair paired-end sequencing was performed using an Illumina MiSeq following manufacturer’s protocols. CASAVA 1.8 was used to perform base calling. The resulting fastq files were used for analysis with QIIME2 (2018.8) (58). Raw sequences were demultiplexed and filtered with q2-deumix plugin followed by denoising with DADA2 (q2-dada2) (123). Amplicons were aligned with mafft (q2-phylogeny) (124). The *de novo* phylogenetic tree from fasttree2 (q2-phylogeny) (125) was used to construct phylogeny trees. The q2-diversity was used to estimate Alpha (Shannon, Chao1, Simpson, and Faith) and Beta-diversity [weighted UniFrac (126)] metrics. To summarize the weighted UniFrac distance matrix, we used a principal coordinate analysis (PCoA). PERMANOVA analysis was performed using QIIME2. The operational taxonomic units (OTUs) were determined by aligning reads to the Greengenes Database (version 13_8) at 99% identity (127). Feature tables describing the relative abundance of bacterial taxa were used for analysis of diversity within each sample. The total levels of bacteria per cecal tip sample were measured by qPCR using eubacterial primers as previously described (15, 60). The absolute abundance of bacterial genera was calculated by multiplying the fraction of total reads for a genus by the total level of bacteria per mg of cecum sample (in arbitrary units).

### Biofilm assays

We used a modified version of the 96-well flat bottom microtiter plate model of *Candida* biofilm formation previously described (65, 128). Briefly, *C. glabrata* was grown overnight in YPD broth at 30°C. Cells were then centrifuged, washed twice in PBS, moved into an anaerobic chamber, and diluted to 1 × 10^6^ cells/mL in pre-reduced DMEM (Corning Cell Gro, Corning, NY, USA cat. MT10-013CV) with 10% heat-inactivated fetal bovine serum (Atlanta Biologicals S11150) and 1% MEM non-essential amino acids (ThermoFisher Scientific, Waltham, MA, USA cat. 11140076). The cell suspension was used to seed wells of a 96-well flat bottom microtiter with 100 µL. Appropriate positive (absence of *C. difficile* to measure *C. glabrata* biofilm formation only) and negative (no cells, to monitor contamination and to be able to calculate percent inhibition) controls were included in each experiment. Plates were incubated anaerobically for 24 h at 37°C to allow for biofilm formation. After 24 h, the media was removed and replaced with fresh media containing *C. difficile* at a concentration of 1 × 10^5^ cells/mL and incubated for a further 24 h. Each experiment was conducted three separate times (biological replicates), with multiple technical replicates per plate. The 2,3-bis-(2-methoxy-4-nitro-5-sulfophenyl)-2*H*-tetrazolium-5-carboxanilide (XTT) assay was specifically used to measure *C. glabrata* viability, and in turn, biofilm formation, in polymicrobial biofilms. After the incubation period following the addition of *C. difficile* or fresh media without *C. difficile*, plates were taken out of the anaerobic chamber, washed twice with 200 µL/well of aerobic PBS to reduce *C. difficile* viability. Biofilm inhibition was measured using the XTT colorimetric assay as previously described (65, 128, 129).

The PrestoBlue cell viability reagent (Invitrogen, Carlsbad, CA, USA cat. A13261) was used to quantify *C. difficile* viability, and in turn, biofilm formation, in polymicrobial biofilms as previously described (130). Briefly, after incubation with or without *C. glabrata*, the supernatant was removed, and samples were washed twice with 200 µL of prereduced PBS. The PBS was removed and 200 µL of 1:10 vol/vol PrestoBlue reagent in DMEM supplemented with 10% FBS and 1% NEAA were added. Plates were then sealed with parafilm, wrapped in foil, and taken out of the anaerobic chamber to incubate for 1 h in a 5% CO_2_ incubator at 37°C. Finally, 100 µL from each well was transferred into a new 96-well plate for fluorescent readings in a plate reader at 560/25 nm excitation and 590/35 emission.

To measure overall biofilm biomass, biofilms were stained with crystal violet as previously described (131). After incubation, plates were washed twice with PBS and 100 µL of methanol was added to each well for 20 min to fix the biofilms. Methanol was removed and plates allowed to dry. Biofilms were then stained for 10 min with 150 µL of 3% (wt/vol) crystal violet. Crystal violet was removed, and plates were allowed to dry. This was followed by three washes with 200 µL of distilled water. Plates were allowed to dry. Each well received 100 µL of 33% glacial acetic acid and was left to de-stain for 5 min while shaking slowly. A total of 85 µL was transferred to a new microtiter plate for OD_550_ measurement. For microscopy, stained samples were directly observed on the 96-well plate using a 20× objective in an inverted system microscope equipped for photography.

### Antifungal and antibacterial susceptibility assays

For antifungal dose response assays, polymicrobial biofilms were formed as described above. After the 24 h incubation with *C. difficile*, media was removed and replaced with media containing twofold dilutions either 5-Flucytosine (Sigma-Aldrich, St. Louis, MO, USA cat. 1272000-200MG), amphotericin B (ThermoFisher Scientific, Waltham, MA, USA cat. MT30003CF), caspofungin (Merck & Co., Inc., Whitehouse Station, NJ), or vancomycin (ThermoFisher Scientific, Waltham, MA, USA cat. BP29581) starting at 200 µg/mL, 1 µg/mL, 16 µg/mL, and 64 µg/mL, respectively. Caspofungin was obtained as a powder and was stored at 4°C; a stock solution was prepared in PBS at 2 mg/mL the same day before its addition to well plates. After anaerobic incubation, the plates were washed twice with 200 µL/well of PBS, and biofilm viability was measured using the XTT and Presto Blue colorimetric assays described above.

### Real-time quantitative PCR analysis of *C. glabrata* gene expression in polymicrobial biofilms

*C. glabrata* RNA was extracted using the Ambion Purelink mini kit (Invitrogen, Carlsbad, CA, USA cat. 12183555). Briefly, media was removed, and biofilms were washed twice in cold PBS. Biofilms were then resuspended in 800 µL of lysis buffer (from kit) containing 1% BME. The resuspended biofilm was then transferred to 2 mL gasketed tubes containing 1.3 mL of zirconium/glass beads. The tubes containing the zirconium/glass beads were stored at −20°C for 30 min before use. Bead beating was conducted thrice in a Biospec Bead Beater 24 for 1 min with a 5 min ice incubation step in between. This was followed by purification with the Ambion Purelink Mini kit. cDNA was synthesized using SuperScript III (Invitrogen, Carlsbad, CA, USA cat. 18080093) as per the manufacturer instructions. qPCR reactions were performed using SYBR Green Master Mix (Applied Biosystems) and a LightCycler 480 II (Roche) instrument. Gene specific primers were previously described (73, 78).

### Statistical analysis

Statistical analyses were performed using GraphPad Prism (San Diego, CA, USA) and Excel.

## Data Availability

The raw sequencing data were submitted in FASTQ format to the NCBI Sequence Read Archive. The BioProject ID is PRJNA918677 and it can be found on https://www.ncbi.nlm.nih.gov/sra.

## SUPPLEMENTAL MATERIAL

Fig S1 msphere.00122-23-s0001.tif *C. glabrata* colonization.

Fig S2 msphere.00122-23-s0002.tif Cecum histology scores.

Fig S3 msphere.00122-23-s0003.tif Cecal microbiota diversity.

Fig S4 msphere.00122-23-s0004.tif Cecal bacterial phyla.

Fig S5 msphere.00122-23-s0005.tif Bacterial abundance.

Fig S6 msphere.00122-23-s0006.tif *C. glabrata* viability.

Fig S7 msphere.00122-23-s0007.tif Biofilms.

Fig S8 msphere.00122-23-s0008.tif Toxin production.

Fig S9 msphere.00122-23-s0009.tif Antifungal dose response.

Fig S10 msphere.00122-23-s0010.tif Vancomycin dose response.

ASM does not own the copyrights to Supplemental Material that may be linked to, or accessed through, an article. The authors have granted ASM a non-exclusive, world-wide license to publish the Supplemental Material files. Please contact the corresponding author directly for reuse.
