# Supplementary figures and images for: Pre-colonization with the fungus Candida glabrata exacerbates infection by the bacterial pathogen Clostridioides difficile in a murine model
Source: mSphere. 2023 Jun 26;8(4):e00122-23. doi: 10.1128/msphere.00122-23 (PMC10449511; doi:10.1128/msphere.00122-23)

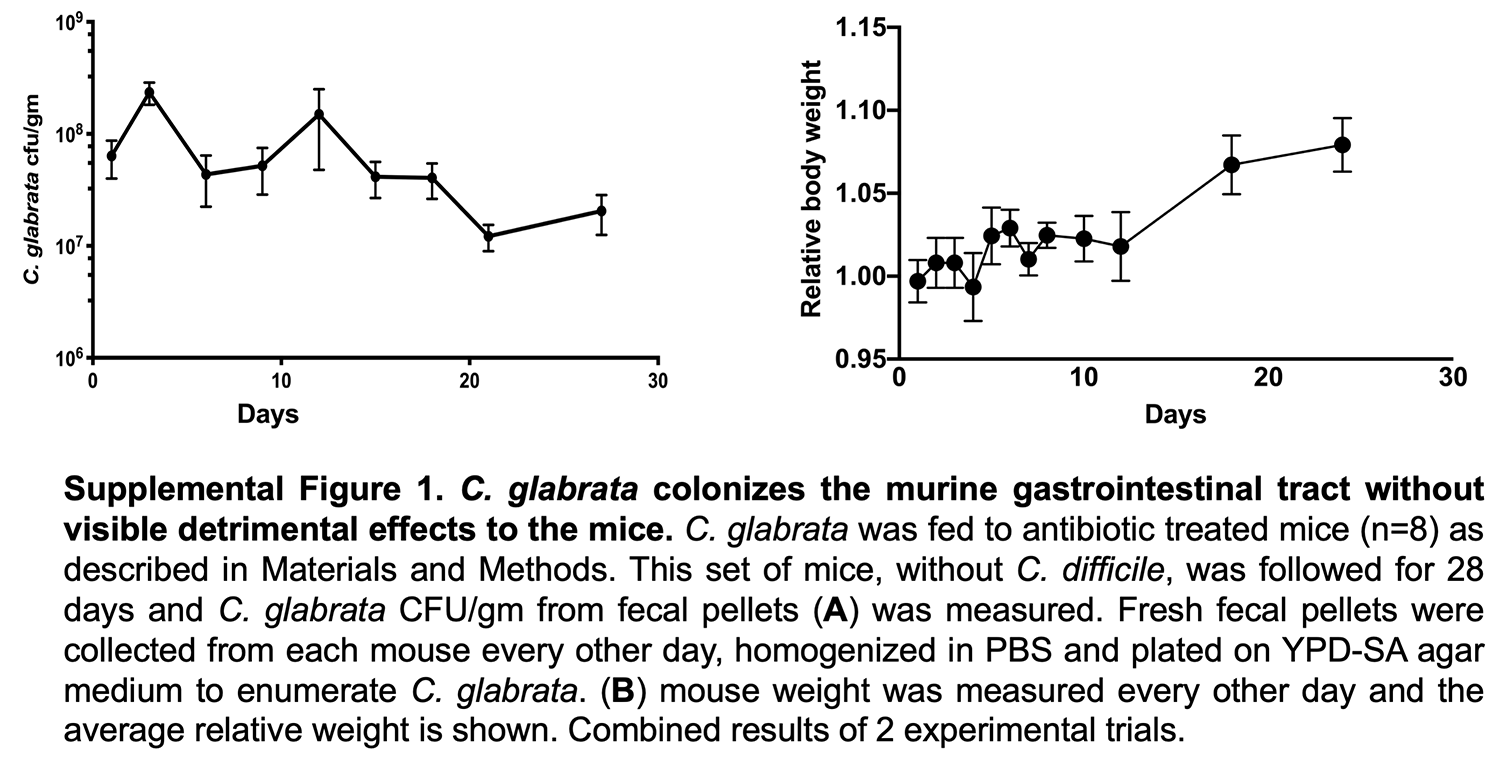

Supplement: Fig S1 — C. glabrata colonization. [file msphere.00122-23-s0001.tif]

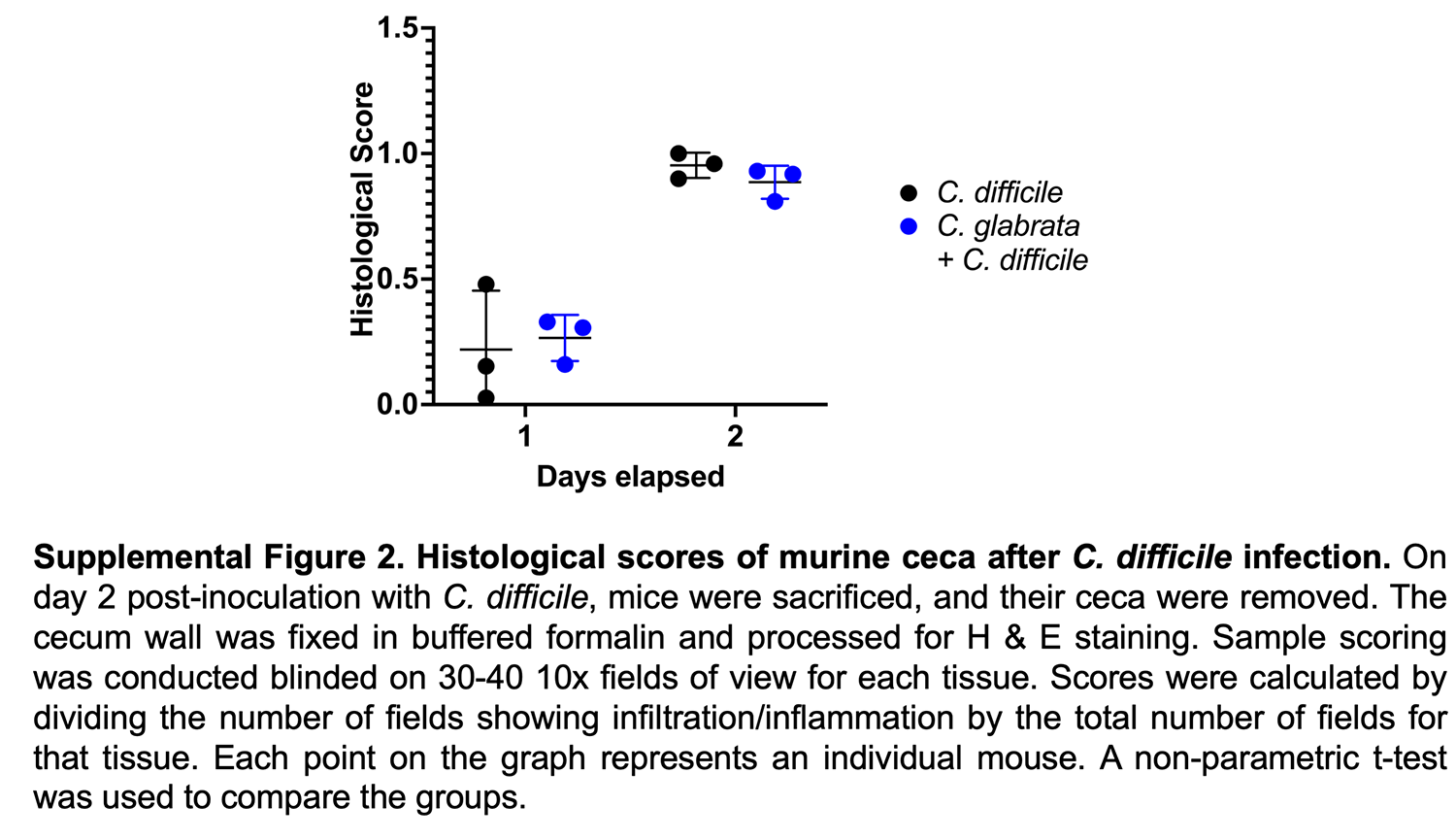

Supplement: Fig S2 — Cecum histology scores. [file msphere.00122-23-s0002.tif]

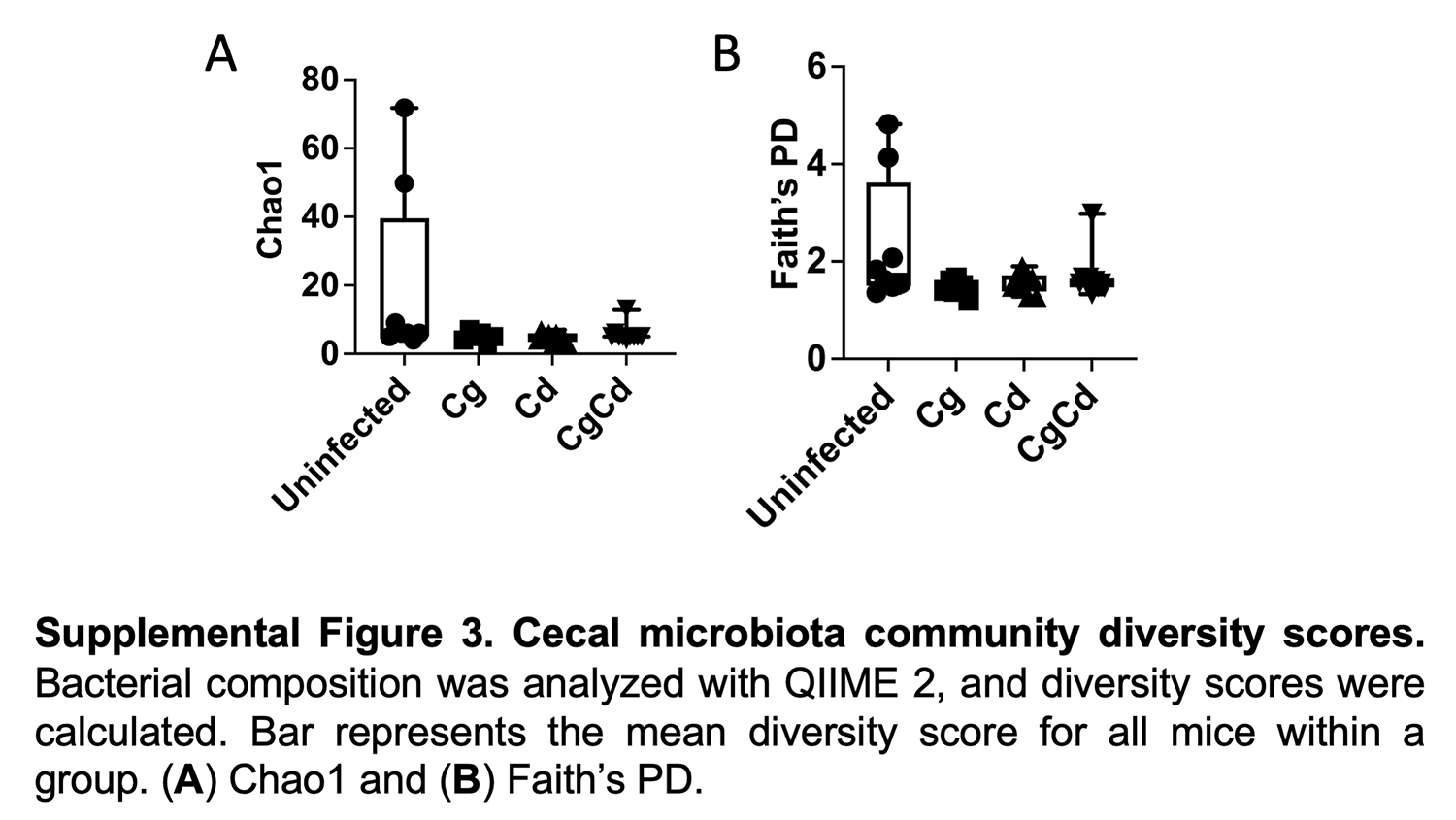

Supplement: Fig S3 — Cecal microbiota diversity. [file msphere.00122-23-s0003.tif]

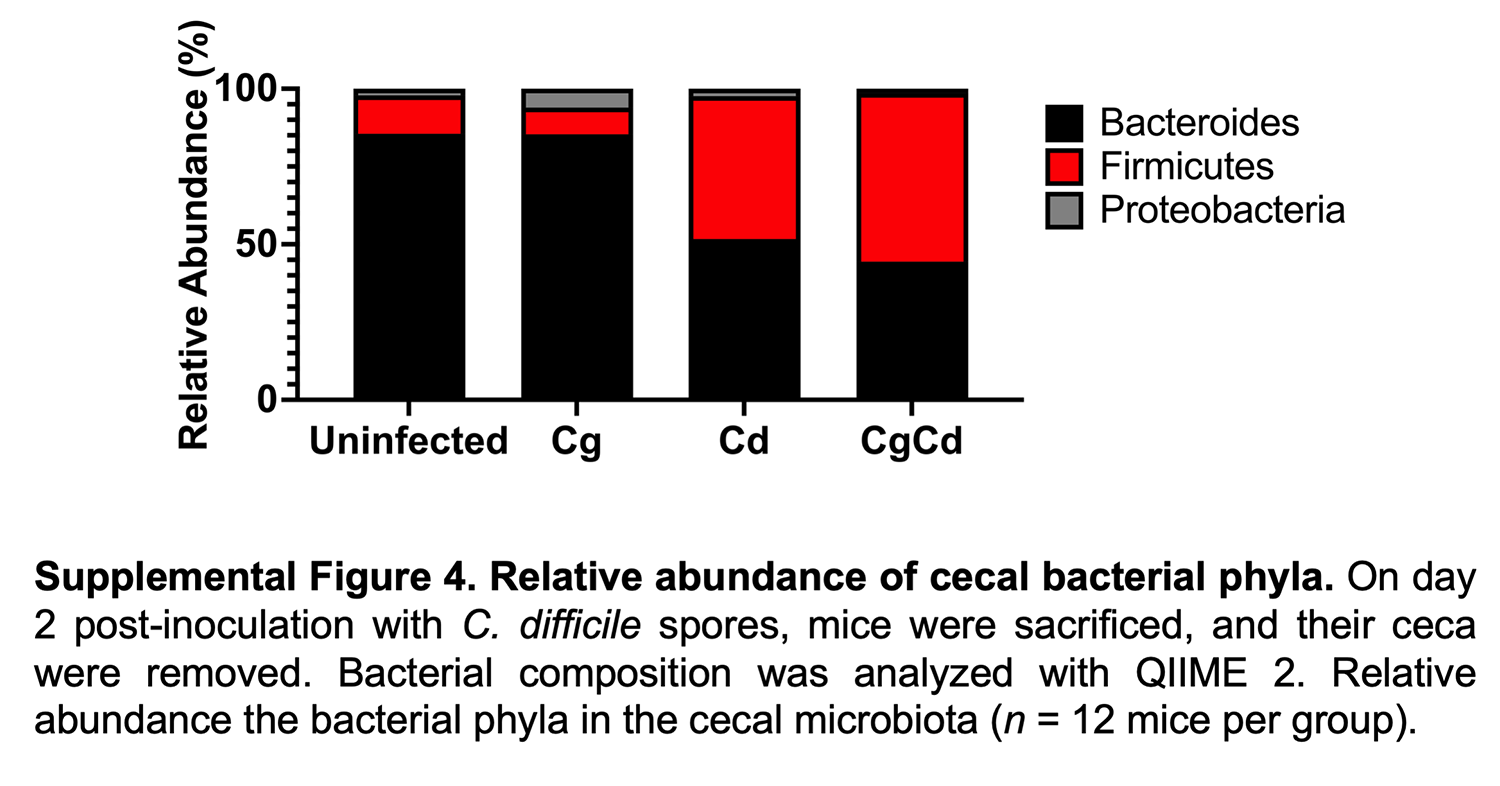

Supplement: Fig S4 — Cecal bacterial phyla. [file msphere.00122-23-s0004.tif]

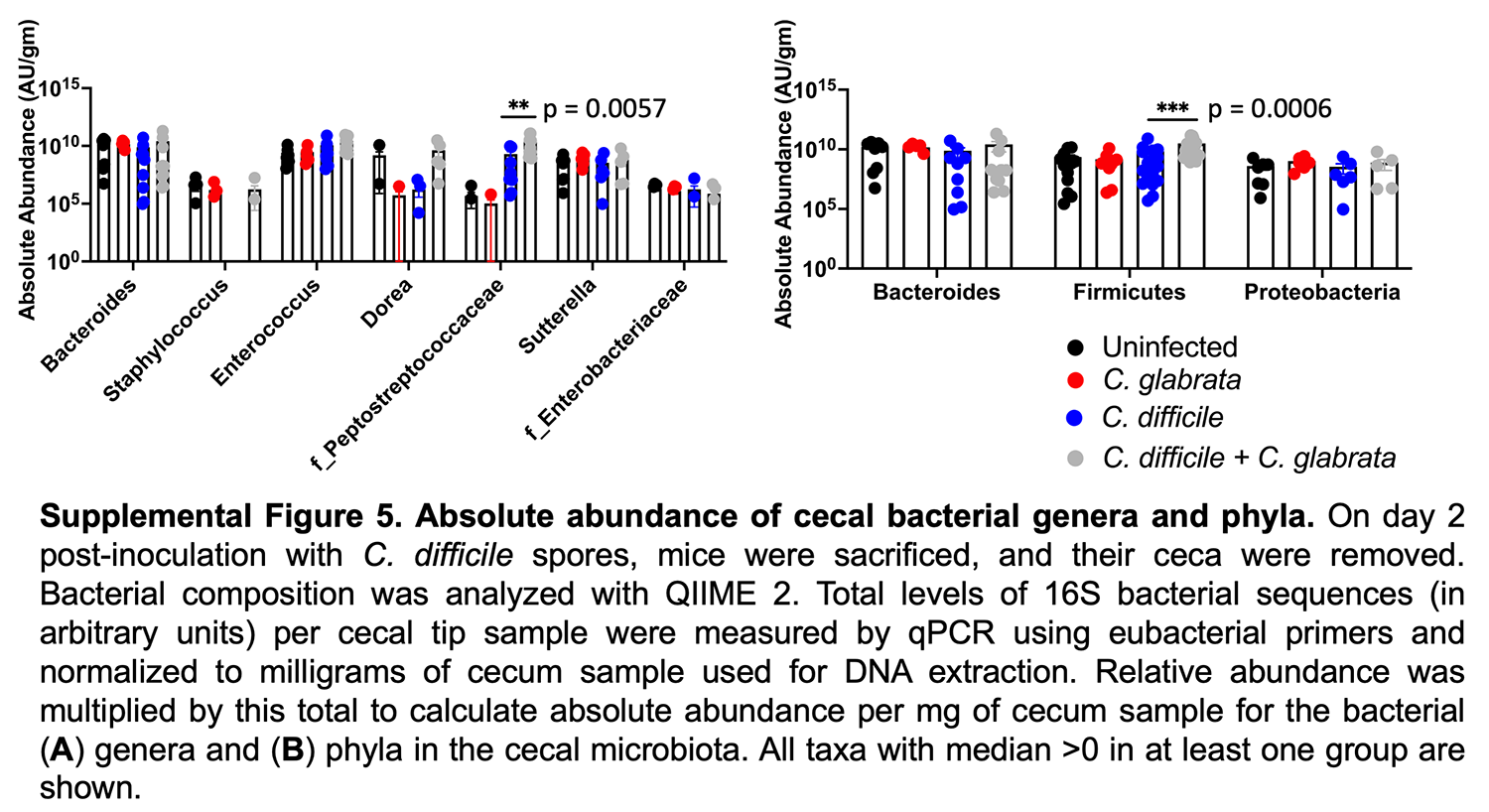

Supplement: Fig S5 — Bacterial abundance. [file msphere.00122-23-s0005.tif]

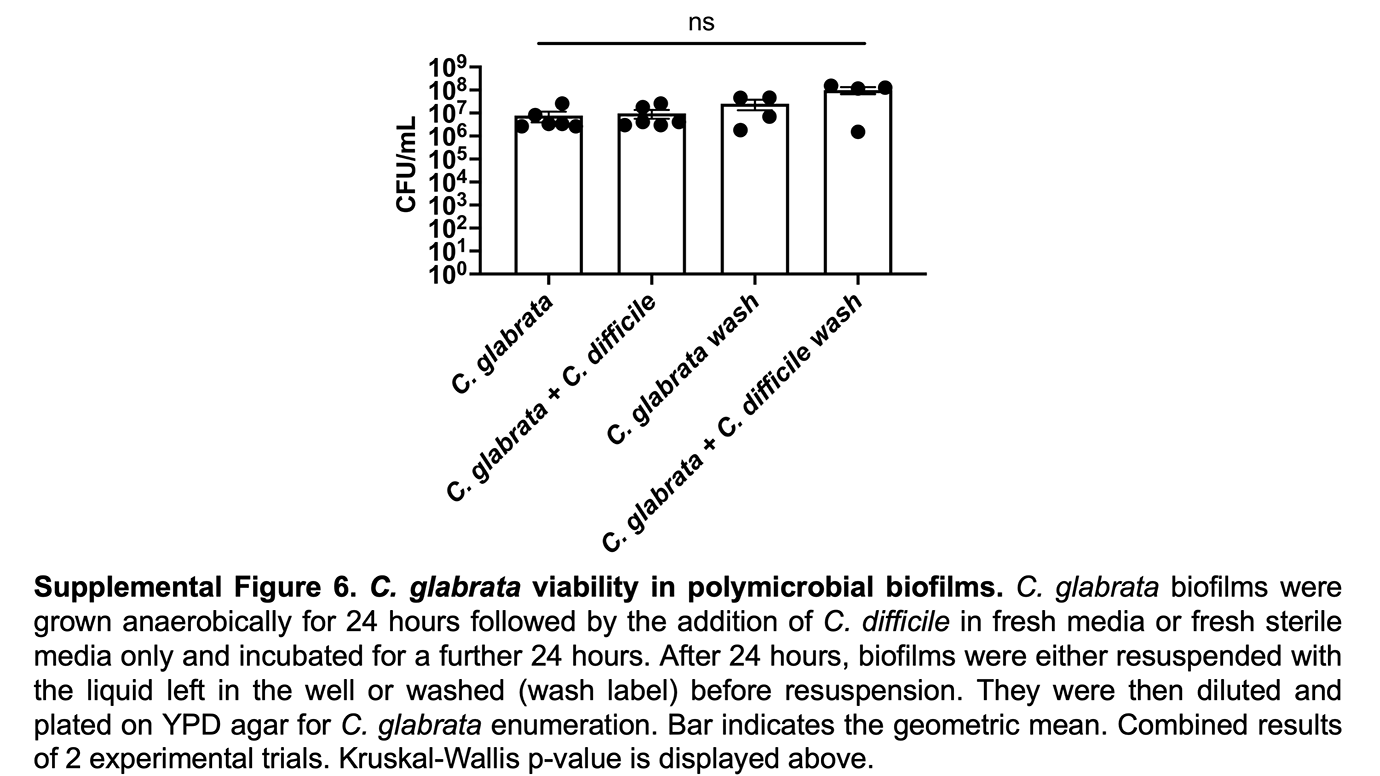

Supplement: Fig S6 — C. glabrata viability. [file msphere.00122-23-s0006.tif]

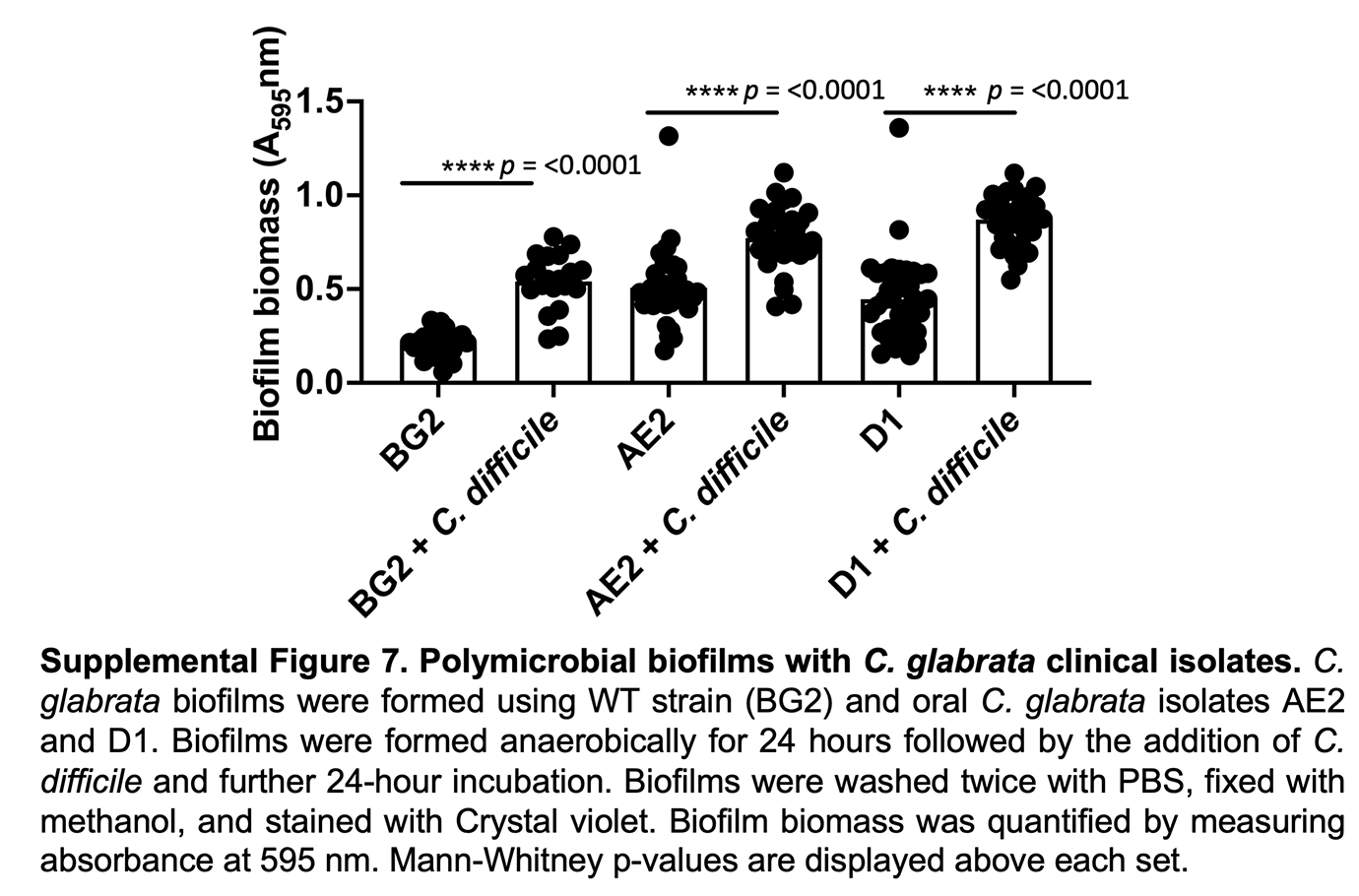

Supplement: Fig S7 — Biofilms. [file msphere.00122-23-s0007.tif]

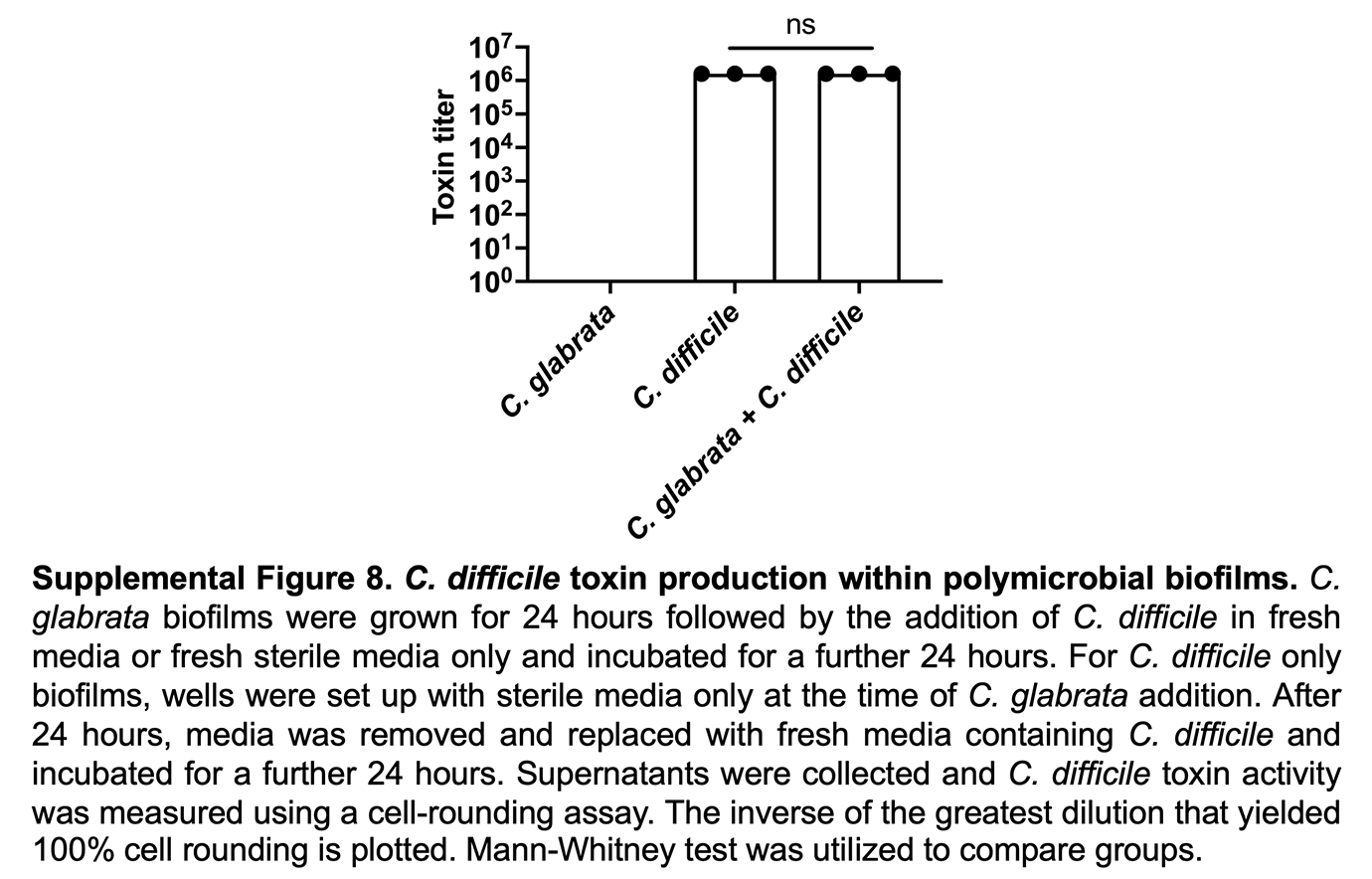

Supplement: Fig S8 — Toxin production. [file msphere.00122-23-s0008.tif]

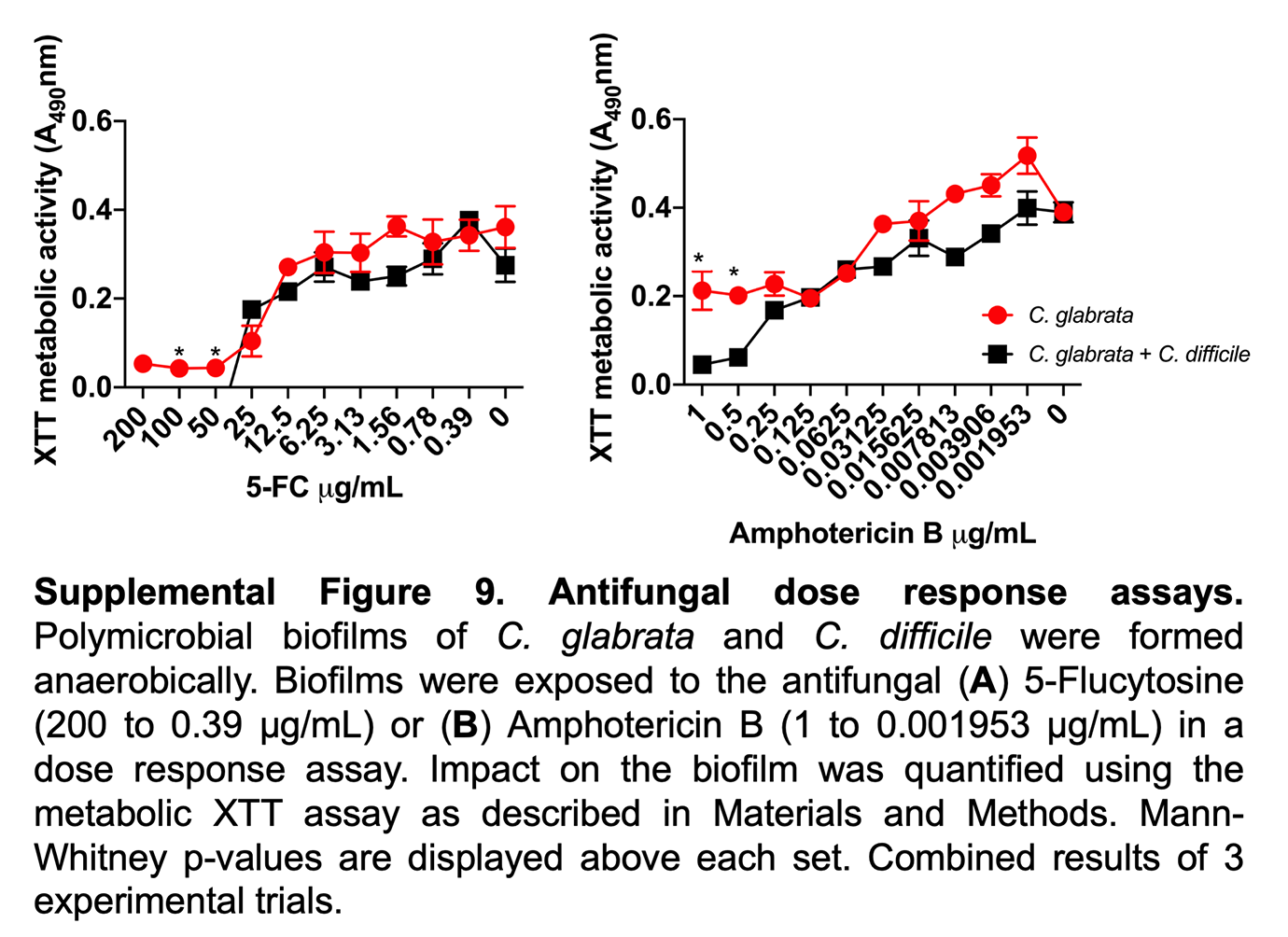

Supplement: Fig S9 — Antifungal dose response. [file msphere.00122-23-s0009.tif]

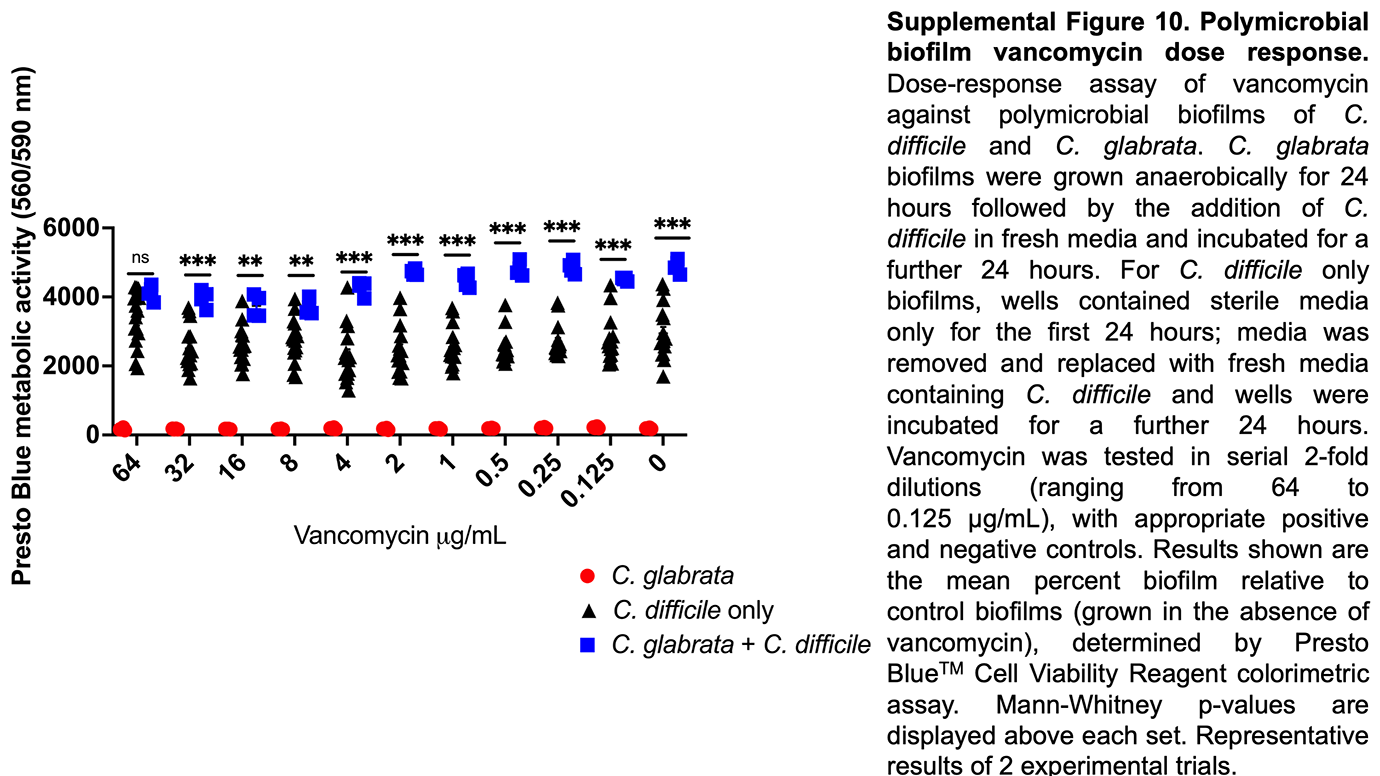

Supplement: Fig S10 — Vancomycin dose response. [file msphere.00122-23-s0010.tif]
